# Clinically important change for the FACIT-Fatigue scale in paroxysmal nocturnal hemoglobinuria: a derivation from international PNH registry patient data

**DOI:** 10.1186/s41687-023-00609-4

**Published:** 2023-07-05

**Authors:** David Cella, Peter Johansson, Yasutaka Ueda, Ioannis Tomazos, Philippe Gustovic, Alice Wang, Ami S. Patel, Hubert Schrezenmeier

**Affiliations:** 1 Department of Medical Social Sciences, Northwestern University Feinberg School of Medicine, 625 N Michigan Ave Suite 2100, Chicago, IL 60611 USA; 2 Sahlgrenska University Hospital, Gothenburg, Sweden; 3 Department of Hematology and Oncology, Osaka University Graduate School of Medicine, Suita, Osaka Japan; 4 Alexion, AstraZeneca Rare Disease, Boston, MA USA; 5 Alexion, AstraZeneca Rare Disease, Zurich, Switzerland; 6 Institute of Transfusion Medicine, University of Ulm, Ulm, Germany; 7 Institute of Clinical Transfusion Medicine, German Red Cross Blood Transfusion Service Baden-Württemberg-Hessen and University Hospital Ulm, Ulm, Germany

## Abstract

**Background:**

Fatigue is the most common symptom associated with paroxysmal nocturnal hemoglobinuria (PNH). The objective of this analysis was to estimate values that would suggest a clinically important change (CIC) for the functional assessment of chronic illness therapy-fatigue scale (FACIT-Fatigue) in patients with PNH.

**Methods:**

Adults with PNH who initiated eculizumab within 28 days of enrollment in the International PNH Registry as of January 2021 with baseline FACIT-Fatigue scores were included in the analysis. Distribution-based estimates of likely difference were calculated using 0.5 × SD and SEM. Anchor-based estimates of CIC considered the European Organization for Research and Treatment of Cancer (EORTC) global health status/quality of life summary score and the EORTC Fatigue Scale score. Changes in anchors and high disease activity (HDA) shift from start of eculizumab treatment to each follow-up visit were then assessed by FACIT-Fatigue score change (≤ 1 CIC, no change, or ≥ 1 CIC).

**Results:**

At baseline, 93% of 423 patients had fatigue documented in their medical history. The distribution-based estimates for FACIT-Fatigue were 6.5 using 0.5 × SD and 4.6 using SEM; internal consistency was high (α = 0.87). For anchor-based estimates, the FACIT-Fatigue CIC ranged from 2.5 to 15.5, and generally supported 5 points as a reasonable lower end of the value for meaningful individual change. The percentage of patients who changed from having HDA at baseline to no HDA at eculizumab-treated follow-up visits increased over time.

**Conclusion:**

These results support the use of 5 points as the CIC for FACIT-Fatigue in patients with PNH, which is within range of the CICs reported in other diseases (3–5 points).

**Supplementary Information:**

The online version contains supplementary material available at 10.1186/s41687-023-00609-4.

## Abstract (plain language summary)

Paroxysmal nocturnal hemoglobinuria (PNH) is a rare blood disease that leads to breakdown of the body’s red blood cells in the blood vessels (intravascular hemolysis). Many people with PNH have fatigue, which consists of tiredness and weakness. A self-report questionnaire called the FACIT-Fatigue scale that objectively measures how fatigued a patient feels has been validated in clinical trials. Changes in the FACIT-Fatigue score help determine if a treatment is helping patients. The clinically important difference (difference between groups of people) or clinically important change (change within an individual) on the FACIT-Fatigue scale is a value that helps patients and clinicians find out if a drug helps or worsens patient fatigue. There is currently no defined clinically important difference and clinically important change on the FACIT-Fatigue scale for people with PNH. The International PNH Registry is a noninterventional, observational study collecting safety, treatment outcomes, and quality of life data from adults with PNH. This study used data from the International PNH Registry to find a clinically important difference and clinically important change in terms of improvement on the FACIT-Fatigue scale for adults with PNH who started eculizumab treatment. Different approaches were used to determine the amount of change in FACIT-Fatigue that would show that eculizumab has meaningful benefits, meaning patients are less fatigued. The authors demonstrated that a 5-point change on the FACIT-Fatigue is meaningful for people with PNH. This number is similar to the clinically important difference and clinically important change values of 3–5 points determined in other diseases.

**Supplementary Information:**

The online version contains supplementary material available at 10.1186/s41687-023-00609-4.

## Introduction

Paroxysmal nocturnal hemoglobinuria (PNH) is a rare, acquired, clonal hematopoietic stem cell disorder characterized by chronic intravascular hemolysis caused by uncontrolled terminal complement activation [1, 2]. Fatigue is the most commonly reported symptom associated with PNH (80% of patients) and is frequently severe [3–5], leading to diminished quality of life (QoL) [6].

The functional assessment of chronic illness therapy-fatigue scale (FACIT-Fatigue) was initially developed in response to the need for a more accurate assessment of fatigue associated with anemia in cancer patients and was later used to evaluate fatigue in other diseases, including PNH [7]. This instrument has been validated in patients with PNH as well as used extensively in clinical trials and in the International PNH Registry [8, 9]. The FACIT-Fatigue scale used in PNH consists of 13 items each scored from 0 to 4, yielding a maximum possible score of 52 points, with higher scores indicative of less fatigue [8].

A clinically important difference (CID; i.e., difference between groups)/clinically important change (CIC; i.e., change within an individual) on the FACIT-Fatigue has not yet been determined for patients with PNH. Studies have therefore generally used the CID estimated for other disease states; for example, a CID of ≥ 3 points for FACIT-Fatigue, calculated for patients with cancer, is frequently used in the studies of patients with PNH [10]. Important differences in FACIT-Fatigue scores between treatment groups in populations with other immune-mediated diseases are estimated to range from approximately 3 to 5 points [11–13].

Eculizumab and ravulizumab are humanized monoclonal antibodies that target C5, a protein of the terminal complement system, and are indicated for the treatment of patients with PNH [14, 15]. Several studies have demonstrated that both eculizumab and ravulizumab significantly alleviate fatigue as indicated by improved FACIT-Fatigue scores [16–21]. In the phase 3 TRIUMPH study, there was significant improvement of ≥ 1 CID in FACIT-Fatigue scores within 1 week of eculizumab treatment [18]. However, the referent ID used in this study was that estimated for patients with cancer. A PNH-specific CIC for FACIT-Fatigue would be informative in interpreting changes in fatigue and could serve as a more robust criterion for evaluating treatment benefit.

Several methods are used to determine important differences and change scores; these approaches are broadly categorized as distribution- or anchor-based methods and each has their strong points and limitations [22]. Distribution-based methods rely on statistical data and use the variability of overall responses to estimate a difference likely to exceed measurement error, but they lack a means to directly evaluate the importance of the change in outcome [22]. Anchor-based approaches use an external indicator (the anchor) to define and quantify importance; however, there is no consensus on what constitutes an appropriate anchor [22]. The US Food and Drug Administration (FDA) recommends the use of multiple anchors that are easy to understand and interpret, as well as sufficiently associated with the clinical outcome assessment (ie, evaluating clinically meaningful change) [23]. Regardless of the approach, estimating the CIC for rare diseases in clinical trials can be challenging because of restrictive sample sizes [24].

The International PNH Registry (NCT01374360) is a prospective, noninterventional, observational study collecting safety, effectiveness, and QoL data from patients with a confirmed PNH diagnosis or detectable PNH clone who are monitored in current medical practice, irrespective of treatment [5]. It is the largest global database of patients with PNH and provides an extensive profile of the progression of PNH and its outcomes. Determining a clinically meaningful improvement in fatigue-related symptoms based on results derived from these data would provide insight into the impact of eculizumab on QoL in a real-world setting. The objective of this analysis was to determine the FACIT-Fatigue CIC for patients with PNH treated with eculizumab using distribution- and anchor-based approaches and real-world data from the International PNH Registry.

## Methods

### Patients

As part of their participation in the International PNH Registry, patients are asked to complete patient-assessment questionnaires regarding health-related QoL, including the FACIT-Fatigue scale, PNH-related symptoms, and health resource utilization. The Registry was approved by the institutional review boards (or equivalent) of participating centers, and all patients provided written informed consent before inclusion. The Registry is sponsored by Alexion, AstraZeneca Rare Disease, and is overseen by an executive committee of internationally recognized PNH medical experts.

For the current analysis, patients at least 18 years of age enrolled in the PNH Registry as of January 18, 2021, with valid patient identification and available date of birth, gender, enrollment date, treatment status, FACIT-Fatigue score at baseline (i.e., date of eculizumab initiation), and at least 1 anchor at baseline were included. Patients had to have initiated eculizumab within 28 days of enrollment in the PNH Registry. Patients also had not received prior treatment with any non-eculizumab anticomplement inhibitor (e.g., ravulizumab) before enrollment in the PNH Registry.

### Outcomes

FACIT-Fatigue is a 13-item self-administered questionnaire; each question provides 5 ordinal response options (“not at all,” “a little bit,” “somewhat,” “quite a bit,” and “very much”) [25]. Each item contributes equally (item score range = 0–4) to a total score that ranges from 0 to 52, where higher scores represent less fatigue. A score will only be calculated if at least 7 of the 13 questions have a response. To properly scale the total score while still accounting for missing values, the sum is prorated by multiplying by 13 and then dividing by the number of answered questions.

FACIT-Fatigue scores were assessed at baseline and at 6, 12, 24, and 36 months (± 3 months) follow-up. When assessing the follow-up visits over 6- and 12-month intervals, the target follow-up dates were calculated forward based on the baseline date of eculizumab initiation. For each of the follow-up time points, the assessment value was selected within a 3-month window before and after the time point of interest. If there were before and after values that were equally close, the before value was selected for analysis.

### Analysis

For patient demographics and clinical history variables, continuous variables were presented using mean, SD, median, (min, max) and/or (Q1, Q3); for categorical variables, frequencies and percentages were presented.

Two distribution-based [26, 27] estimates were calculated using the following: 0.5 × SD and SEM. The SEM was calculated as SD × sqrt (1–α), where α represents the internal consistency coefficient Cronbach’s α. Cronbach’s α was calculated from the 13 FACIT-Fatigue items.

Clinical outcome variables considered as anchors (predefined category) included the number of red blood cell (RBC) transfusion units within the 12 months before starting anti-complement therapy (i.e., C5 inhibitors; 0 units; > 1 unit[s]) and the following multiple-item outcomes: the European Organisation for Research and Treatment of Cancer (EORTC) Global Health Status/QoL summary score (quartiles) and the EORTC fatigue scale score (quartiles), which are subscales found within the EORTC Quality of Life Questionnaire (EORTC QLQ-C30) [29]. A higher EORTC Global Health Status/QoL score indicates better QoL, and a higher EORTC Fatigue Scale score indicates more fatigue [28]. To assess the appropriateness of the proposed clinical outcomes as anchors, Spearman’s correlation between each outcome and the FACIT-Fatigue score was performed at baseline. Outcomes with a correlation whose absolute value was > 0.30 were used as anchors.

A cross-sectional approach was used to estimate the anchor-based CID. For each of the anchors, the mean baseline FACIT-Fatigue score was first calculated within their respective predefined category (e.g., within quartiles for the EORTC variables). Subsequently, the mean differences in mean FACIT-Fatigue score between adjacent categories were calculated, and this final mean was referenced as the anchor-based CID. After the baseline important difference measurements were calculated, the 2 distribution-based estimates were assessed in conjunction with the anchor-based mean differences.

FACIT-Fatigue was then classified at each time point using the referent CIC, defined as the distribution-based estimate most consistent (i.e., high α value) with the anchor-based mean changes. Patients were classified into 1 of 3 groups based on the change in the FACIT-Fatigue score at each follow-up visit relative to baseline: (1) FACIT-Fatigue score increased by at least 1 CIC; (2) FACIT-Fatigue score was within 1 CIC (this group was the referent in the following analysis); and (3) FACIT-Fatigue score decreased by at least 1 CIC. For assessment of anchor-based changes in FACIT-Fatigue, change from baseline was calculated for each of the continuous variables at each follow-up visit. To determine the FACIT-Fatigue change from baseline at each follow-up visit by EORTC Global Health Status/QoL score and EORTC Fatigue score groupings, patients were classified into 1 of 3 groups based on the change in the EORTC score at each follow-up visit relative to baseline: (1) EORTC score increased by at least 1 IC; (2) EORTC score was within 1 IC (this group was the referent in the analysis); and (3) EORTC score decreased by at least 1 IC. Change from baseline was subsequently calculated for FACIT-Fatigue at each follow-up visit. Means, SDs, medians, and Q1 and Q3 for each change variable were presented according to the EORTC-anchor grouping at each time point. Nonparametric testing was used to analyze differences in change from baseline across the FACIT-Fatigue or EORTC-anchor groups, using a 0.05 α level.

Changes in anchors and high disease activity (HDA) shift (“yes” to “no” from baseline to each follow-up visit) were then assessed by FACIT-Fatigue score change (≤ 1 CIC, no change, or ≥ 1 CIC). HDA was defined as LDH ratio ≥ 1.5 × upper limit of normal (ULN) and at least 1 of the following: history of a major adverse vascular event (including a thrombotic event); anemia defined by hemoglobin < 10 g/dL; or physician-reported abdominal pain, dyspnea, dysphagia, fatigue, hemoglobinuria, or erectile dysfunction at any time before the date of interest. Finally, the distribution of the HDA shift for each of the 2 groups with at least 1 CIC change in FACIT-Fatigue was compared to the referent group using a χ^2^ test. Frequencies and percentages were presented, along with a χ^2^ *P* value. Those patients with missing data necessary for the classification of HDA were excluded.

## Results

As of January 18, 2021, there were 5549 patients enrolled in the International PNH Registry. Of these, 5016 patients had valid patient identification, enrollment date, date of birth, sex and eculizumab treatment status at enrollment. The analysis population comprised 423 patients with available FACIT-Fatigue score and at least 1 available clinical anchor at baseline, age ≥ 18 years old at initiation of eculizumab (within 28 days of enrollment in the Registry), and untreated with any non-eculizumab complement inhibitor at enrollment. The demographics of the study population (Table 1) were similar to those of the overall International PNH Registry population who were excluded from the study (n = 5126; Additional file 1: Table S1). Among patients excluded from the study with eculizumab treatment status (n = 1593, Additional file 1: Table S1), 54% were female, 79% were white or of Caucasian descent, the mean age at PNH start was 37.0 years, and the mean age at baseline (i.e., initiation of eculizumab) was 43.6 years. Of the 423 patients included in the study, 53% were female, the majority were white or of Caucasian descent (84%), and mean ages at PNH start and at baseline (i.e., initiation of eculizumab) were 39.0 and 45.9 years, respectively (Table 1). At baseline, 93% of patients in the study had physician documentation of fatigue in their medical history; the mean FACIT-Fatigue score was 29.4 for patients in the study and 31.1 for patients excluded from the study.

**Table 1:** Patient demographics and baseline disease characteristics

Characteristic	Patients (N = 423)
Sex, n (%)	
Female	226 (53)
Male	197 (47)
Race, n (%)	
White or Caucasian descent	355 (84)
Asian	51 (12)
Black or African descent	9 (2)
Other (unlisted, multiple races, Aboriginal)	8 (2)
Age at PNH start,^a^ year	
Mean ± SD	39.0 ± 17.5
Median (Q1, Q3)	35.0 (23.2, 51.6)
Age at baseline,^b^ year	
Mean ± SD	45.9 ± 17.2
Median (Q1, Q3)	44.4 (31.7, 60.2)
Mean ± SD baseline hemoglobin,^c^ g/dL	9.7 ± 2.1
Mean baseline LDH ratio × ULN,^d^ n (%)	
< 1.5	40 (11)
≥ 1.5	324 (89)
Percent GPI-deficient granulocytes,^e^ n (%)	
< 10%	5 (2)
≥ 10% to < 50%	35 (11)
≥ 50%	282 (88)
Physician-documented fatigue,^f^ n (%)	
Yes	386 (93)
No	31 (7)
Mean ± SD baseline FACIT-Fatigue score	29.4 ± 12.9

*FACIT* functional assessment of chronic illness therapy, *GPI* glycosylphosphatidylinositol, *LDH* lactate dehydrogenase, *PNH* paroxysmal nocturnal hemoglobinuria, *ULN* upper limit of normal

^a^PNH start date is defined as the earliest date among the following: PNH diagnosis, date of first PNH symptoms, and/or date of reported granulocytes clone lab text

^b^Baseline was defined as the date of eculizumab initiation

^c^Baseline hemoglobin n = 370

^d^Baseline LDH n = 364

^e^GPI = deficient granulocytes, n = 322

^f^Physician-documented fatigue, n = 417. Values are mean (%) unless otherwise noted

### Clinically important differences and clinically important changes

Distribution-based estimates for FACIT-Fatigue were 6.5 when using 0.5 × SD and 4.6 when using SEM (Table 2). Internal consistency was high, with Cronbach’s α = 0.87; hence, the SEM-based value was selected as the referent CID. The correlations between FACIT-Fatigue score and clinical anchors that met the predetermined baseline threshold > 0.3 were as follows: 0.73 for EORTC Global Health Status/QoL Scale, and − 0.86 for EORTC Fatigue Scale; these outcomes were therefore used as anchors [30]. For these anchor-based measurements, the CID of FACIT-Fatigue was 8.4 using the EORTC Global Health Status/QoL Scale score and 9.5 using the EORTC Fatigue Scale score (Table 3).

**Table 2:** Important difference of FACIT-Fatigue score at baseline

	n	Mean FACIT-Fatigue score	r	σ	Important difference	
					0.5 × σ	SEM
Total population	423	29.4	0.873	12.9	6.5	4.6

*FACIT* functional assessment of chronic illness therapy, *r* reliability (Cronbach's alpha)

**Table 3:** Important difference of FACIT-Fatigue by EORTC Global Health Status/QoL score and EORTC Fatigue scale

	EORTC Global Health Status/QoL score^c^				
	Q1 (n = 90)	Q2 (n = 98)	Q3 (n = 111)	Q4 (n = 120)	Difference (range)
Mean FACIT-Fatigue Score^a^	15.7	25.5	31.1	40.9	8.4 (5.6–9.8)
*P* value^b^					< 0.0001

	EORTC Fatigue scale score^d^				
	Q1 (n = 84)	Q2 (n = 117)	Q3 (n = 96)	Q4 (n = 121)	Difference (range)
Mean FACIT-Fatigue Score^a^	44.8	36.0	24.4	16.3	− 9.5 (− 8.1 to − 11.6)
*P* value^b^					< 0.0001

*EORTC* European Organization for the Research and Treatment of Cancer, *FACIT* functional assessment of chronic illness therapy, *QoL* quality of life

^a^Higher FACIT-Fatigue scores indicate less fatigue

^b^*P* value is calculated using the analysis of variance (ANOVA)

^c^Increasing quartiles for EORTC Global Health Status/QoL indicate higher functioning

^d^Increasing quartiles for EORTC Fatigue Scale indicate more fatigue

**Fig. 1:**
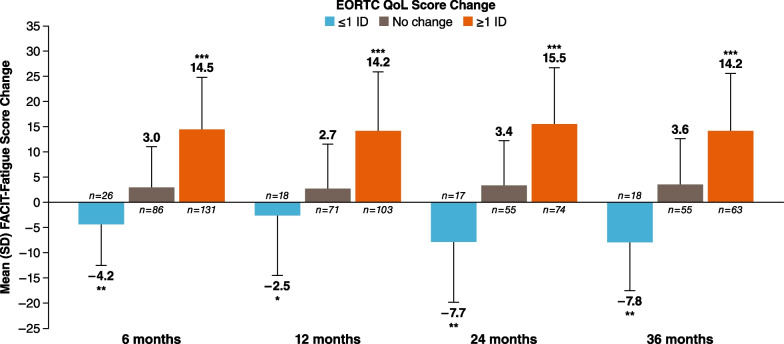
Mean FACIT-Fatigue change from baseline to follow-up by EORTC Global Health Status/QoL score grouping. EORTC QoL, European Organization for Research and Treatment of Cancer Quality of Life; FACIT, Functional Assessment of Chronic Illness Therapy; IC, important change. *P < 0.05, **P < 0.001, ***P < 0.0001

**Fig. 2:**
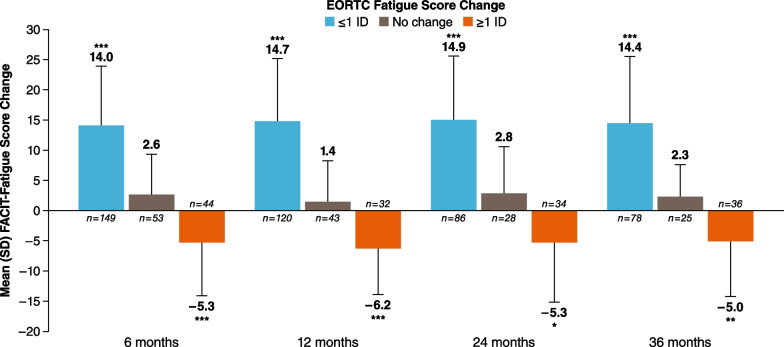
Mean FACIT-Fatigue change from baseline to follow-up by EORTC Fatigue score grouping. EORTC, European Organization for Research and Treatment of Cancer; FACIT, Functional Assessment of Chronic Illness Therapy; IC, important change. *P < 0.05, **P < 0.001, ***P < 0.0001

Starting at 6 months, patients experienced a significant improvement in their mean (SD) FACIT-Fatigue score from baseline by EORTC Global Health Status/QoL score grouping that was sustained at each follow-up visit (≤ 1 IC: − 4.2 [8.2], *P* < 0.001; no change: 3.0 [8.1]; ≥ 1 IC: 14.5 [10.3], *P* < 0.0001 at 6 months; Fig. 1). The statistically significant changes for EORTC Global Health Status/QoL score ranged from 2.5 to 15.5. There was a significant decrease in the mean (SD) FACIT-Fatigue score from baseline by EORTC Fatigue score at the 6-month follow-up visit (≤ 1 IC: 14.0 [9.9], *P* < 0.0001; no change: 2.6 [6.7]; ≥ 1 IC: − 5.3 [8.8], *P* < 0.0001), which was sustained through the 36-month follow-up visit (Fig. 2). Significant changes in EORTC Fatigue scores ranged from 5.0 to 14.9.

### Outcomes by CIC in FACIT-Fatigue

**Fig. 3:**
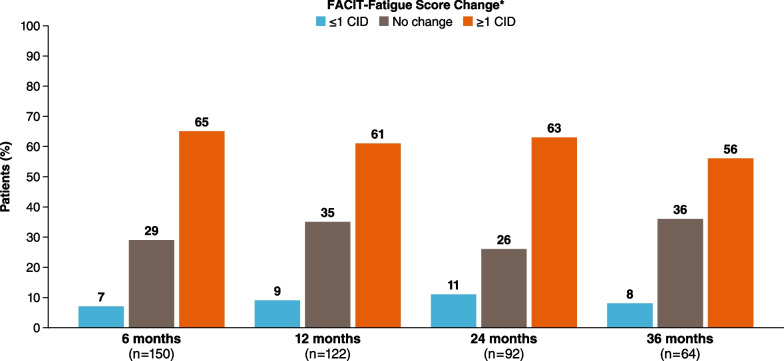
Change in percentage of patients with HDA at baseline and no HDA during follow-up by FACIT-Fatigue score change. CIC, clinically important change; FACIT, Functional Assessment of Chronic Illness Therapy; HDA, high disease activity (defined as lactate dehydrogenase ≥ 1.5 × upper limit of normal and ≥ 1 PNH symptom); PNH, paroxysmal nocturnal hemoglobinuria. *CIC reflects the SEM-derived value of 5 points

The percentage of patients who changed from having HDA at baseline to no HDA at eculizumab-treated follow-up visits increased over time. Using the SEM value of 4.6 as the referent CIC, most of these patients (65%) experienced ≥ 1 CIC in FACIT-Fatigue at 6 months that was sustained through 36 months (Fig. 3). Results were similar when 0.5 × SD was used; with this CIC, the proportion of patients who experienced ≥ 1 CIC in FACIT-Fatigue was 62% at 6 months, 58% at 12 months, 60% at 24 months, and 55% at 36 months.

## Discussion

To the best of our knowledge, this is the first study to estimate a clinically important change for FACIT-Fatigue in patients with PNH. A CIC of 5 points is suggested for FACIT-Fatigue in patients with PNH. Highly reliable tests such as the FACIT-Fatigue (Cronbach’s α = 0.87) produce SEM values that are lower than the 0.5 × SD criterion. While a 5-point change was identified as the starting point for CIC, anchor-based results indicate that values as low as 3 points could be considered. In this case, the SEM-based estimate of 5 points is justified over the SD-based estimate, owing to the reliability of the FACIT-Fatigue. This estimate is close to the range of important differences (3–5 points) reported for diseases including cancer (CID = 3) [10]; hematologic diseases such as cold agglutinin disease (CID = 5) [31]; and immune-related diseases such as moderate to severe psoriatic arthritis (CID = 3.1) [13], rheumatoid arthritis (CID = 3–4) [11], and systemic lupus erythematosus (3- to 7-point change for distribution- and anchor-based estimates of minimally important difference) [12].

To determine the CIC, distribution-based methods use the variability of overall responses and rely on statistical data [22]. Distribution-based methods do not compare changes in the scores of patient-reported outcome measures (i.e., patient’s perspective not considered) and therefore do not incorporate any measure to reflect the importance of the change in outcome. They may be calculated as several different values based on assumptions made (e.g., related to effect size or degree of variability in the baseline scores of the patient-reported outcome measure) [32].

The anchor-based approach uses external criteria (the anchor) to determine the clinical significance of the degree of change in scores (i.e., to classify patients as improved or worsened and link it to the score difference), but these are not necessarily the *minimally* meaningful differences [33]. Anchor-based methods also incorporate the patient’s perspective and are often the preferred approach to estimating CIC [34]. However, with anchor-based methods, the anchor question may not fully capture changes in a patient-reported outcome measure that may reflect more than 1 type of outcome. It is also not clear how much change in the anchor itself is clinically important. Moreover, there may be recall bias because patients may not accurately recollect their pretreatment status at the posttreatment time point when the anchor is applied [32]. Therefore, it is important that the interpretation and application of a patient-reported outcome measure’s CIC is relevant to and reflective of the patient population to which it is being applied (e.g., similar clinical context, disease severity, and treatment given). A low CIC value may overestimate the positive effects of the treatment, whereas a high CIC value may misclassify patients as being nonresponsive to treatment, when the treatment was in fact helpful. A strength of this analysis, therefore, was the use of the large International PNH Registry to provide an extensive real-world profile of PNH disease course and outcomes.

In this study approach, patients were placed into 4 distinct groups as defined by the EORTC scores, and the differences between the 4 groups were estimated. The differences are meaningful so long as correlation and significance criteria are met, but caution is required to not assume that the differences are “minimally” important. Limitations of this study include that the analysis was based on an observational data set and not all patients had available data for every outcome assessed, which may limit the applicability of the study findings to the general population. However, demographics of the study population were similar to those of the overall Registry population, suggesting that the estimated CIC may be relevant to and reflective of individuals with PNH. Additionally, the results were based on patients treated with eculizumab and while they may be generalizable to PNH patients receiving other treatments, this assumption would benefit from future research.

## Conclusions

These findings support the use of 5 points as the CIC for FACIT-Fatigue in individual patients with PNH. Although a CIC of 5 may not be the minimal value, it is close to the range of CIDs reported in other diseases (3–5 points) [10–13, 31]. This finding, obtained from a real-world data set with a large number of patients, helps establish an important metric for assessment of a meaningful treatment response of patients with PNH.

## Supplementary Information

**Additional file 1: Table S1**. Demographics and Baseline Disease Characteristics.

## Data Availability

Alexion, AstraZeneca Rare Disease will consider requests for disclosure of clinical study participant-level data provided that participant privacy is assured through methods such as data de-identification, pseudonymization, or anonymization (as required by applicable law), and if such disclosure was included in the relevant study informed-consent form or similar documentation. Qualified academic investigators may request participant-level clinical data and supporting documents (statistical analysis plan and protocol) pertaining to Alexion-sponsored studies. Further details regarding data availability and instructions for requesting information are available in the Alexion Clinical Trials Disclosure and Transparency Policy at http://alexion.com/our-research/research-and-development. Link to data-request form: https://alexion.com/contact-alexion/medical-information.

## Abbreviations

CIC: Clinically important change
CID: Clinically important difference
EORTC: European Organization for Research and Treatment of Cancer
FACIT: Functional assessment of chronic illness therapy
FDA: Food and drug administration
HDA: High disease activity
IC: Important change
LDH: Lactate dehydrogenase
PNH: Paroxysmal nocturnal hemoglobinuria
QoL: Quality of life
RBC: Red blood cell
ULN: Upper limit of normal
