# Supplementary material for: Clinically important change for the FACIT-Fatigue scale in paroxysmal nocturnal hemoglobinuria: a derivation from international PNH registry patient data
Source: J Patient Rep Outcomes. 2023 Jul 5;7:63. doi: 10.1186/s41687-023-00609-4 (PMC10322798; doi:10.1186/s41687-023-00609-4)
Supplement: Supplementary file 1 — Additional file 1: Table S1. Demographics and Baseline Disease Characteristics. [file 41687_2023_609_MOESM1_ESM.docx]

**Supplementary Table**

Table S1. Demographics and Baseline Disease Characteristics (Excluded Patients)

| **Characteristic** | Excluded Patients with Eculizumab Treatment Status  (N=1593) | All Excluded Patients  (N=5126) |
| --- | --- | --- |
| Sex,^a^ n (%) |  |  |
| Female | 851 (54) | 2665 (53) |
| Male | 741 (47) | 2415 (48) |
| Race,^b^ n (%) |  |  |
| White or Caucasian descent | 1254 (79) | 3825 (76) |
| Asian | 211 (13) | 932 (18) |
| Black or African descent | 68 (4) | 156 (3) |
| Other (unlisted, multiple races, Aboriginal, Pacific Islander) | 50 (3) | 144 (3) |
| Age at PNH start,^c^ y |  |  |
| Mean ± SD | 37.0±17.4 | 40.3±18.8 |
| Median (Q1, Q3) | 32.8 (23.5, 48.5) | 36.6 (24.6, 55.0) |
| Age at baseline,^d^ y |  |  |
| Mean ± SD | 43.6±17.1 | 43.6±17.1 |
| Median (Q1, Q3) | 41.1 (29.8, 56.5) | 41.1 (29.8, 56.5) |
| Mean ± SD baseline hemoglobin,^e^ g/dL | 9.3±1.9 | 9.3±1.9 |
| Mean baseline LDH ratio × ULN,^f^ n (%) |  |  |
| <1.5 | 99 (10) | 99 (10) |
| ≥1.5 | 895 (90) | 895 (90) |
| Mean ± SD baseline FACIT-Fatigue score^g^ | 31.1±13.6 | 31.1±13.6 |

FACIT, Functional Assessment of Chronic Illness Therapy; LDH, lactate dehydrogenase; PNH, paroxysmal nocturnal hemoglobinuria; ULN, upper limit of normal.

^a^Excluded patients, n=5080; excluded patients with eculizumab treatment status, n=1592.

^b^Excluded patients, n=5057; excluded patients with eculizumab treatment status, n=1583.

^c^PNH start date is defined as the earliest date among the following: PNH diagnosis, date of first PNH symptoms, and/or date of reported granulocytes clone lab text. PNH start date: excluded patients, n= 5081; excluded patients with eculizumab treatment status, n=1593.

^d^Baseline was defined as the date of eculizumab initiation. Both excluded patients and excluded patients with eculizumab treatment status, n=1593.

^e^Baseline hemoglobin: excluded patients and excluded patients with eculizumab treatment status, n=1132.

^f^Baseline LDH: excluded patients and excluded patients with eculizumab treatment status, n=994.

^g^Baseline FACIT-Fatigue score: excluded patients and excluded patients with eculizumab treatment status, n=15.

Values are mean (%) unless otherwise noted.
